# Effect-Size Discrepancies in Literature Versus Raw Datasets from Experimental Spinal Cord Injury Studies: A CLIMBER Meta-Analysis

**DOI:** 10.1089/neur.2024.0038

**Published:** 2024-07-16

**Authors:** Emma G. Iorio, Alireza Khanteymoori, Kenneth A. Fond, Anastasia V. Keller, Lex Maliga Davis, Jan M. Schwab, Adam R. Ferguson, Abel Torres-Espin, Ralf Watzlawick

**Affiliations:** ^1^ Department of Neurological Surgery, University of California, San Francisco, California, USA.; ^2^ Department of Neurosurgery, Medical Center, Faculty of Medicine, University of Freiburg, Freiburg, Germany.; ^3^ Departments of Neurology and Neurosciences, The Ohio State University, Columbus, Ohio, USA.; ^4^ Belford Center for Spinal Cord Injury, The Ohio State University, Columbus, Ohio, USA.; ^5^ San Francisco Veterans Affairs Healthcare System, San Francisco, California, USA.; ^6^ Department of Physical Therapy, University of Alberta, Edmonton, Canada.; ^7^ School of Public Health Sciences, University of Waterloo, Waterloo, Canada.

**Keywords:** individual animal data (IAD), literature-extracted data (LED), meta-analysis, meta-science, spinal cord injury (SCI), systematic review

## Abstract

Translation of spinal cord injury (SCI) therapeutics from pre-clinical animal studies into human studies is challenged by effect size variability, irreproducibility, and misalignment of evidence used by pre-clinical versus clinical literature. Clinical literature values reproducibility, with the highest grade evidence (class 1) consisting of meta-analysis demonstrating large therapeutic efficacy replicating across multiple studies. Conversely, pre-clinical literature values novelty over replication and lacks rigorous meta-analyses to assess reproducibility of effect sizes across multiple articles. Here, we applied modified clinical meta-analysis methods to pre-clinical studies, comparing effect sizes extracted from published literature to raw data on individual animals from these same studies. Literature-extracted data (LED) from numerical and graphical outcomes reported in publications were compared with individual animal data (IAD) deposited in a federally supported repository of SCI data. The animal groups from the IAD were matched with the same cohorts in the LED for a direct comparison. We applied random-effects meta-analysis to evaluate predictors of neuroconversion in LED versus IAD. We included publications with common injury models (contusive injuries) and standardized end-points (open field assessments). The extraction of data from 25 published articles yielded *n* = 1841 subjects, whereas IAD from these same articles included *n* = 2441 subjects. We observed differences in the number of experimental groups and animals per group, insufficient reporting of dropout animals, and missing information on experimental details. Meta-analysis revealed differences in effect sizes across LED versus IAD stratifications, for instance, severe injuries had the largest effect size in LED (standardized mean difference [SMD = 4.92]), but mild injuries had the largest effect size in IAD (SMD = 6.06). Publications with smaller sample sizes yielded larger effect sizes, while studies with larger sample sizes had smaller effects. The results demonstrate the feasibility of combining IAD analysis with traditional LED meta-analysis to assess effect size reproducibility in SCI.

## Introduction

Spinal cord injury (SCI) affects up to 580,000 new patients worldwide every year and an estimated 1.5–5.2 million patients are suffering from the consequences of SCI.^1,2^ Despite rising optimism,^3^ no pharmaco-biological interventions are widely effective in human SCI. Translation of SCI therapeutics from pre-clinical animal studies into humans is limited by fundamental disconnects in how scientific the pre-clinical and the clinical literature weigh evidence.^4–6^ Pre-clinical results are judged based on the biological novelty of findings and elegance of the methods, whereas clinical findings focus on the stability of findings in the face of clinical variation. In clinical research, systematic reviews and meta-analyses demonstrating reproducible therapeutic effects are considered the highest quality of evidence (class-I evidence), representing a major tool for weighing evidence for clinical decision support.^7^ Conversely, pre-clinical literature values primary data analysis over secondary analysis to assess reproducibility. The lack of reproducibility testing in pre-clinical literature has led some clinical researchers to question the translational value of animal models for predicting clinical trial outcomes.^8^ Yet, clinical translation in SCI is relatively new^1^ and depends on sustained pioneering efforts to drive ongoing bidirectional “translational dialogue.”^9–11^ Aligning the evidentiary basis of pre-clinical and clinical findings represents a fundamental gap, bridgeable through meta-science.

The overall goal of the present study is to assess the feasibility of developing class-I evidence in pre-clinical research through meta-analysis. The SCI community has pioneered efforts to recover individual animal data (IAD) from inaccessible article records (“dark data”),^12^ making them findable, accessible, interoperable, and reusable (FAIR) for translational decision support in the Open Data Commons for SCI (ODC-SCI).^13,14^ This opens the possibility of meta-analysis including literature-extracted data (LED) and IAD analyzed in parallel. Applying meta-analytical methodologies to animal studies to identify, appraise, select, and synthesize all available high-quality research evidence remains a challenging task. Pioneering efforts by the Collaborative Approach to Meta-Analysis and Review of Animal Data in Experimental Studies (CAMARADES) group starting in the early 2000s focused on pre-clinical stroke research.^15,16^ CAMARADES methods have been extended to numerous neurological disease models, including SCI.^17–19^

Heterogeneity and reporting biases within pre-clinical studies have been proposed to contribute to the translational disconnect of pre-clinical and clinical SCI research.^18^ The ARRIVE (Animal Research: Reporting of *In Vivo* Experiments) guidelines provide a checklist to articulate dropout of animals because of morbidity/mortality; however, this practice is often not followed.^20,21^ Statistical tools that detect when subjects have been selectively removed from published analyses (publication bias) suggest that data on “dropout” in animal studies lead to substantial overstatement of efficacy in SCI experiments by up to 40.9%.^18^ In clinical literature, “loss-to-follow-up” is a common problem that is acknowledged, reported, and statistically managed. This inflation of effect sizes because of publication bias is common across fields.^22^ One limitation of literature-based meta-analysis is that statistical tools used to infer missing data rely on data estimable from published literature, in which author curation and editorial decision shaped the nature of the data selected for inclusion. With the rise of data-sharing mandates from funders and journals, it is now possible to directly analyze individual data points undergirding the published literature.^23^ Hence, investigating individual data represent an opportunity to directly quantify the impact of publication bias and relevance of noncurated data and its impact on effect size.

**FIG. 1.**
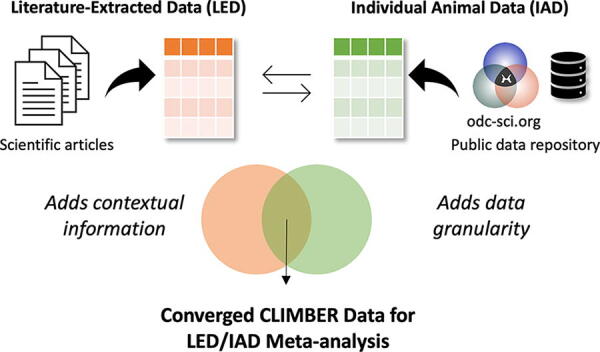
Diagram representing the process of CLIMBER meta-analysis. Converging data from public data repositories (odc-sci.org) and scientific articles allows for the unique meta-analysis done by CLIMBER.

In this study, we performed LED and IAD meta-analysis to study the following: (1) the effects missing data have on published findings, (2) predictors of significant functional improvement (neuroconversion) in animals from numerous studies, and (3) feasibility of benchmarking translation across pre-clinical research (Fig. 1).

## Methods

### Search and selection of publicly available articles

Articles were selected through a methodical review of publications with the corresponding IAD publicly accessible within ODC-SCI^13^ (odc-sci.org, RRID:SCR_016673) as of October 2021 and the VISION-SCI data repository.^24^ As of April 2024, the ODC-SCI is the only National Institutes of Health (NIH)-supported specialist repository that complies with federal data management and sharing policies.^25^ When publicly available raw data on subjects are posted on ODC-SCI, information about corresponding article publication is listed under “Provenance and Originating Publications” on the dataset landing page. Using this information, we successfully linked the datasets to their published literature by using the availability of PubMed identifiers and publication information posted with datasets in ODC-SCI. To prepare for a comparable and reproducible analysis, publications needed to meet the following criteria: (1) have available corresponding IAD, (2) be a pre-clinical SCI study, (3) include neurobehavioral outcomes with time points, (4) report the number of animals per experimental group, and (5) report the mean effect size and its variance (standard deviation or standard error of the mean). All articles included one of three animal species (mice, rat, nonhuman primates). After selecting articles for inclusion, we confirmed that our IAD matched with LED shown in the selected peer-reviewed article by taking advantage of the fact that the Open Data Commons for Traumatic Brain Injury (ODC-TBI) system requires data uploaders to report unique subject identifiers and experimental groups for each subject. This allowed us to compare the reported sample sizes for each group from the published article to the IAD and cross-reference subject groupings and align IAD to LED.

### Data extraction from publications

Two independent reviewers systematically extracted values from the figures and tables from published articles. When data points were only expressed graphically, we used WebPlotDigitizer (v.4.5) to extract data. Data points expressed serially were recorded numerically. On some occasions, information was impossible to extract because of poor plot quality or digitization issues (e.g., pseudo-3D plots with distorted proportionality). We included every time point for which all experimental groups had an outcome score. Additional categorical variables were collected for subgroup analyses. Datasets from two independent reviewers were merged and reviewed to eliminate errors. Discrepancies were adjudicated through supervised discussions by a third reviewer. The final dataset included data and information from all selected articles with many variables, including the following: animal age, species, sex, outcome measure, outcome scale, injury details, author information, and publication information. The full LED analysis dataset is publicly available at the odc-sci.org (RRID:SCR_016673; https://dx.doi.org/10.34945/F5DG6D).

### Identification of IAD

After extraction of data from published articles, corresponding IAD were exported from ODC-SCI and combined with VISION-SCI. Datasets contained different approaches for expressing identical information, such as different labels and units (i.e., time postoperation was reported in both days and weeks). To ensure consistency and facilitate meaningful comparisons, data sets were merged, and variables harmonized to consistent units to standardize diverse data elements across the unified dataset. To minimize the potential for human error, the process of merging and harmonizing this dataset was executed exclusively within the *tidyverse* package in R version 4.3.0.^26,27^ The full IAD dataset for analysis is publicly available at the odc-sci.org (RRID:SCR_016673; https://dx.doi.org/10.34945/F5J59P).

### Inclusion criteria for meta-analysis

Stringent inclusion criteria helped manage heterogeneity between studies. We only included publications that featured data from common injury models and standardized end-points. As contusive injuries made up 48.6% (895/1841) of LED, we excluded publications reporting other injury models. Included publications reported at least one of the following assessment measures: the Basso, Beattie, and Bresnahan Locomotor Scale (BBB Score),^28^ Grooming Test,^29^ Forelimb Open Field Scores,^30^ the Basso Mouse Scale (BMS Score), and BMS Subscores.^31^ Each study included neurobehavioral recovery scores over time. Variations between the durations of behavioral assessments prompted selection of specific time points for analysis to establish a more uniform time frame across the different studies. Studies were included if they reported outcome assessment scores obtained between 0 and 3 days postoperative (DPO) and again at 42 DPO (±14 DPO). There were no inclusion or exclusion criteria related to study intervention. In the IAD, we were sometimes unable to determine which experimental group a subject belonged to because of missing values or lack of information. Subjects with undetermined experimental grouping were excluded.

### Analysis

To compare diverse outcomes, we used standardized mean difference (SMD) as the effect size metric, in accordance with meta-analysis guidelines.^32^ Standardized effect size allows for comparable results across studies with different outcome scales and measurement. We selected outcome measures that were closest to 1 DPO (within 0 and 3 days) serving as baseline and 42 DPO (within 28 and 56 days) to determine the respective effect for each experimental group. To derive standardized effect sizes for this percent change, we calculated SMD for paired samples using the formula from Borenstein^32^:

(1) $$d=\frac{{\bar{Y}}_{1}-{\bar{Y}}_{2}}{S_{\mathrm{within}}}$$

where $\bar{Y_{1}}$ is the outcome score mean of each group at our chosen end-point (*∼*42 DPO) and $\bar{Y_{2}}$ is the outcome score mean of each group at our baseline point (*∼*1 DPO). *S_within_* was calculated as follows:

(2) $$S_{\mathrm{within}}=\frac{S_{di\;f\;f}}{\sqrt{2(1-r)}}$$

where *r* is the correlation between the baseline and effect at ∼42 DPO and *S_diff_* was calculated using the standard deviation (*SD*) of both respective groups as follows:

(3) $$s_{di\;f\;f}=\sqrt{SD_{1}^{2}+SD_{2}^{2}-2\cdot r\cdot SD_{1}\cdot \;SD_{2}}$$

The variance of *d* was calculated using the following:

(4) $$V_{d}=(\frac{1}{n}+\frac{d^{2}}{2n})2(1-r)$$

And the standard error of *d* was calculated using the square root of *V_d_*.

SMD effect size is a standardized statistical entity, reflecting the degree of improvement relative to the baseline. This standardized measure enables us to compare diverse end-points and therapies using a consistent benchmark.

A random-effects meta-analysis was performed to calculate an overall estimate of effect size, using the metagen function from the *meta* package in R with a significance level set at α = 0.05.^32–34^ The Restricted Maximum Likelihood estimation method was used to estimate the additive (between-study) component of variance *τ*^2^ with studies weighted based on the inverse variance of effect sizes and the Hartung–Knapp (HK) adjustment was applied for random-effects models.^35^ To analyze the pooled outcomes, we used 95% confidence interval (CI). The Cochran Q test and the Higgins *I^2^* test were used to assess heterogeneity between the outcomes of the various studies. The dependent variable was the SMD in all cases. Subsequently, regression analyses were performed to examine the impact of subgroups using the metabind function from the *meta* package in R.^33^ The following factors were tested: animal type, animal sex, animal strain, injury severity, injury level, and sample sizes on SMD recovery at that last time point. Using the metafor package in R, we generated forest plots to display our outcomes graphically.^36^ The statistical approaches are described in greater detail elsewhere.^32,35^

## Results

### Literature search and selection

**FIG. 2.**
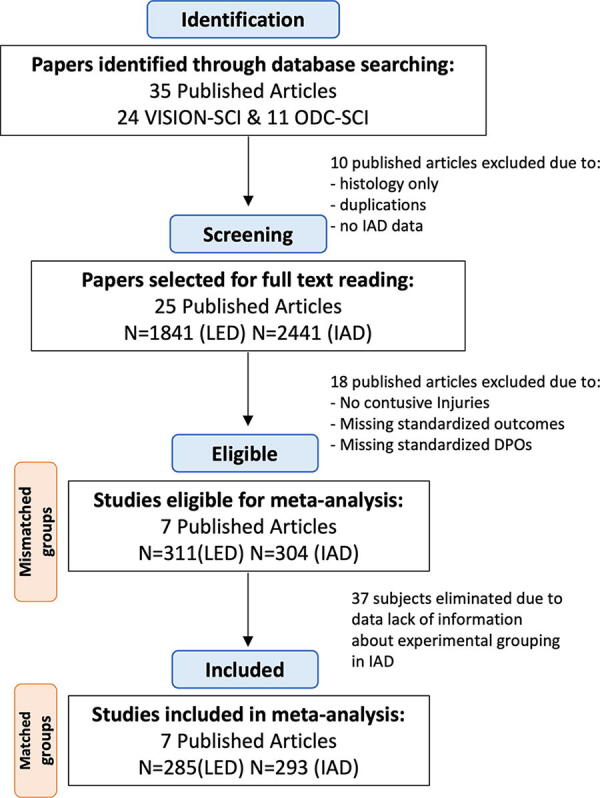
Flowchart illustrating the process of literature screening and selection. The initial search identified 35 articles with corresponding data in ODC-SCI and VISION-SCI. Ten of these articles were excluded for various reasons, which resulted in 25 articles with *n* = 1841 for literature-extracted data and *n* = 2441 for subject-level data. Inclusion criteria allowed for 7 eligible publications with *n* = 311 subjects (25 experimental groups) for literature-extracted data and *n* = 304 subjects (21 experimental groups) for individual animal data. Groups and subjects with lack of information were eliminated because of the inability to match subjects from literature-extracted data to individual animal data. Final inclusion resulted in *n* = 285 (21 experimental groups) animals for literature-extracted data and *n* = 293 (20 experimental groups) for individual animal data. ODC, open data commons; SCI, spinal cord injury.

An initial search and data selection are shown in Figure 2. The initial search identified 11 articles from the ODC-SCI (Table 1) and 24 articles from the VISION-SCI data set (Table 2), accumulating to a total of 35 publications. Of these publications, 10 were excluded because of the following: histology only (no function), unavailable comparison data, duplicated studies, and studies with incomparable data (bioenergetics and review articles). This resulted in 25 included publications with *n* = 1841 for LED and *n* = 2441 for IAD. After applying inclusion criteria, 7 eligible publications were included for LED (*n* = 311 subjects; 25 experimental groups; (Supplementary Table S1) and IAD (*n* = 304 subjects; 21 experimental groups; (Supplementary Table S2). We then performed individual experimental group matching aligning IAD and LED. In the IAD, there were instances where the experimental grouping for some subjects was not reported, which impaired our ability to directly compare with the animals in the LED as we were unable to determine their appropriate cohort. Owing to these incomplete data and insufficient information, 37 subjects were excluded. Our final analysis resulted in 21 experimental groups for LED (*n* = 285 animals, Table 3) and 20 experimental groups for IAD collated from the VISION-SCI database and uploaded to the ODC-SCI repository (*n* = 293; Table 4). We observed misalignment of the numbers of experimental study groups in two cases, and differences in the reported scores and differences in the number of animals per group.

**Table 1.** Corresponding ODC-SCI DOI Numbers of Evaluated Articles

PMID	Dataset DOI number	ODC-SCI accession #	Dataset title	Contact	Date published
23544088	http://doi.org/10.7295/W9T72FMZ	26	Cervical (C5), unilateral spinal cord injury with diverse injury modalities, multiple behavioral outcomes, and histopathology	Adam Ferguson	November 4, 2018
26466022	http://doi.org/10.34945/F5V884	567	A comprehensive set of hemodynamic parameters, blood gases and hemoglobin levels during spinal cord injury procedure followed by weekly assessments of locomotor and bladder function recovery with survival histology data from 334 rats in the MASCIS trial	Adam Ferguson	March 3, 2021
28817801	http://doi.org/10.7295/W9HQ3X20	212	T10 lateral hemisection spinal cord injury with multiple histological and behavioral outcomes	Zhigang He	August 19, 2019
31786212	http://doi.org/10.34945/F59885	458	Behavior and histopathology after single-session cortical electrical stimulation and rehabilitative forelimb motor training in cervical spinal cord injury in females rats	Karim Fouad	November 17, 2020
31940312	http://doi.org/10.34945/F5XW2P	578	Effects of a fecal transplant from anxious donors on rehabilitative training, microbiota composition, systemic inflammation and behaviour following a unilateral cervical spinal contusion (C5, 125 kdyn) in female Lewis rats	Karim Fouad	April 8, 2021
32647037	http://doi.org/10.34945/F54S3W	432	Repeated measurements of hindlimb CatWalk variables in normal rats	Nick Jeffery	August 13, 2020
32735618	http://doi.org/10.34945/F5QP4H	419	Data for manuscript: Delayed short-term tamoxifen treatment does not promote remyelination or neuron sparing after spinal cord injury	Dana M. McTigue	June 24, 2020
33290776	http://doi.org/10.34945/F5ZW20	553	Self-directed reaching and grasping rehabilitation using automatic pellet presentation system after cervical dorsolateral quadrant injury in female rats	Fenrich Keith	January 14, 2021
33651310	http://doi.org/10.34945/F5QG66	595	Hemodynamics, weight and overground locomotion from 1125 rats with different spinal cord injury thoracic contusion severities recovered from the Multicenter Spinal Cord Injury Study (MASCIS)	Adam Ferguson	March 3, 2021
34174901	http://doi.org/10.34945/F5F30N	454	Minocycline treatment for acute cervical spinal cord injury in female rats: microbiota composition	Karim Fouad	January 22, 2021
31940312	http://doi.org/10.7295/W97942VQ	262	Data for the manuscript: Fecal Transplant Prevents Gut Dysbiosis and Anxiety-like Behaviour After Spinal Cord Injury in Rats	Karim Fouad	December 3, 2020

ODC, open data commons; SCI, spinal cord injury.

**Table 2.** Evaluated Articles from VISION-SCI Data Repository

PMID	DOI number	Publication title	Authors	Year published
8986744	https://doi.org/10.1038/nm0197-73	Apoptosis and delayed degeneration after spinal cord injury in rats and monkeys.	Crowe, M et al.	1997
9417825	https://doi.org/10.1006/exnr.1997.6695	Endogenous repair after spinal cord contusion injuries in the rat	Beattie, MS et al.	1997
9418967	https://doi.org/10.1002/(SICI)1097–4547 (19971201)50:5<798::AID-JNR16>3.0.CO;2-Y	Apoptosis of microglia and oligodendrocytes after spinal cord contusion in rats	Shuman, SL et al.	1998
9624630	https://doi.org/10.1089/neu.1998.15.451	External anal sphincter hyperreflexia following spinal transection in the rat	Holmes, GM et al.	2009
11402879	https://doi.org/10.1177/154596830001400405	An analysis of changes in sensory thresholds to mild tactile and cold stimuli after experimental spinal cord injury in the rat	Lindsey, AE et al.	2000
12440371	https://doi.org/10.1016/S0079-6123(02)37019-5	Spinal cord contusion models	Young, W	2002
12675971	https://doi.org/10.1089/08977150360547099	Experimental Modeling of Spinal Cord Injury: Characterization of a Force-Defined Injury Device	Scheff, SW et al.	2004
12908927	https://doi.org/10.1089/089771503322144572	Creatine diet supplement for spinal cord injury: influences on functional recovery and tissue sparing in rats	Rabchevsky, AG et al.	2004
15473991	https://doi.org/10.1016/j.expneurol.2004.06.029	Quantitative assessment of deficits and recovery of forelimb motor function after cervical spinal cord injury in mice	Anderson, KD et al.	2004
15530870	https://doi.org/10.1016/j.expneurol.2004.05.043	Acute transplantation of glial-restricted precursor cells into spinal cord contusion injuries: survival, differentiation, and effects on lesion environment and axonal regeneration	Hill CE et al.	2004
15899253	https://doi.org/10.1016/j.expneurol.2005.02.006	Quantitative assessment of forelimb motor function after cervical spinal cord injury in rats: Relationship to the corticospinal tract	Anderson, KD et al.	2005
16430371	https://doi.org/10.1089/neu.2006.23.36	Behavioral and Histological Characterization of Unilateral Cervical Spinal Cord Contusion Injury in Rats	Gensel JC et al.	2006
17115911	https://doi.org/10.1089/neu.2006.23.1654	The Louisville Swim Scale: A Novel Assessment of Hindlimb Function following Spinal Cord Injury in Adult Rats	Smith, RR et al.	2006
17603042	https://doi.org/10.1016/j.expneurol.2007.05.024	Spinal pathways involved in the control of forelimb motor function in rats	Anderson, KD et al.	2007
19331515	https://doi.org/10.1089/neu.2008.0829	Swim training initiated acutely after spinal cord injury is ineffective and induces extravasation in and around the epicenter	Smith, RR et al.	2009
19733168	https://doi.org/10.1016/j.expneurol.2009.08.020	Forelimb locomotor assessment scale (FLAS): Novel assessment of forelimb dysfunction after cervical spinal cord injury	Anderson, KD et al.	2009
19886808	https://doi.org/10.1089/neu.2009.0914	Gait Analysis in Normal and Spinal Contused Mice Using the TreadScan System	Beare, JE et al.	2009
20302862	https://doi.org/10.1016/j.expneurol.2010.03.008	Task-specificity vs. ceiling effect: Step-training in shallow water after spinal cord injury	Kuerzi, J et al.	2010
21168495	https://doi.org/10.1016/j.nbd.2010.12.010	CD47 knockout mice exhibit improved recovery from spinal cord injury	Myers, SA et al.	2011
21963672	https://doi.org/10.1016/j.expneurol.2011.09.023	The PPAR alpha agonist gemfibrozil is an ineffective treatment for spinal cord injured mice	Almad, A et al.	2011
22445934	https://doi.org/10.1016/j.neuroscience.2012.03.006	Acetyl-l-carnitine treatment following spinal cord injury improves mitochondrial function correlated with remarkable tissue sparing and functional recovery	Patel, SP et al.	2012
8654527	https://doi.org/10.1006/exnr.1996.0098	Graded Histological and Locomotor Outcomes after Spinal Cord Contusion Using the NYU Weight-Drop Device versus Transection	Basso, DM et al.	1996
21076427	https://doi.org/10.1038/nn.2691	Extensive spontaneous plasticity of corticospinal projections after primate spinal cord injury	Rosenzweig, E et al.	2010
22331214	https://doi.org/10.1177/1545968311421934	Methods for Functional Assessment After C7 Spinal Cord Hemisection in the Rhesus Monkey	Nout, YS et al.	2012

SCI, spinal cord injury.

**Table 3.** Summary of Experimental Groups from Literature-Extracted Data

PMID	Cohort label	Neurobehavioral score	Sample size	Animal type	Injury level	Sex	Strain
11402879	12.5 mm	BBB score	4	Rat	Thoracic	Female	Long–Evans
	25.0 mm	BBB score	14	Rat	Thoracic	Female	Long–Evans
	6.25 mm	BBB score	4	Rat	Thoracic	Female	Long–Evans
12675971	100kdyn	BBB score	10	Rat	Thoracic	Female	Sprague–Dawley
	150kdyn	BBB score	9	Rat	Thoracic	Female	Sprague–Dawley
	200kdyn	BBB score	8	Rat	Thoracic	Female	Sprague–Dawley
16430371	12.5 mm	Grooming score	11	Rat	Cervical	Female	Long–Evans
	6.25 mm	Grooming score	10	Rat	Cervical	Female	Long–Evans
21963672	Study I: Vehicle	BMS score	5	Mice	Thoracic	Female	C57BL/6J
	Study II-Pre: Drug	BMS score	6	Mice	Thoracic	Female	C57BL/6J
	Study II-Pre: Vehicle	BMS score	6	Mice	Thoracic	Female	C57BL/6J
22445934	Drug	BBB score	14	Rat	Lumbar	Female	Sprague–Dawley
	Vehicle	BBB score	14	Rat	Lumbar	Female	Sprague–Dawley
23544088	100kdyn	Grooming score	34	Rat	Cervical	Female	Long–Evans
	12.5 mm	Grooming score	32	Rat	Cervical	Female	Long–Evans
	6.25 mm	Grooming score	10	Rat	Cervical	Female	Long–Evans
	75kdyn	Grooming score	58	Rat	Cervical	Female	Long–Evans
32735618	Drug: Female	BMS score	10	Mice	Thoracic	Both	C57BL/6J
	Drug: Male	BMS score	9	Mice	Thoracic	Both	C57BL/6J
	Vehicle: Female	BMS score	8	Mice	Thoracic	Both	C57BL/6J
	Vehicle: Male	BMS score	9	Mice	Thoracic	Both	C57BL/6J

BBB, Basso, Beattie, and Bresnahan Locomotor Scale; BMS, Basso mouse scale open-field score.

**Table 4.** Summary of Experimental Groups from Individual Animal Data

PMID	Cohort label	Neurobehavioral score	Sample size	Animal type	Injury level	Sex	Strain
11402879	12.5 mm	BBB score	4	Rat	Thoracic	Female	Long–Evans
	25.0 mm	BBB score	14	Rat	Thoracic	Female	Long–Evans
	6.25 mm	BBB score	4	Rat	Thoracic	Female	Long–Evans
12675971	100kydn	BBB score	16	Rat	Thoracic	Female	Sprague–Dawley
	150kydn	BBB score	15	Rat	Thoracic	Female	Sprague–Dawley
	200kydn	BBB score	12	Rat	Thoracic	Female	Sprague–Dawley
16430371	12.5 mm	Forelimb OpenField score	11	Rat	Cervical	Female	Long–Evans
	6.25 mm	Forelimb OpenField score	6	Rat	Cervical	Female	Long–Evans
21963672	Study I: Vehicle	BMS score	5	Mice	Thoracic	Female	C57BL/6J
	Study II-Pre: Drug	BMS score	7	Mice	Thoracic	Female	C57BL/6J
	Study II-Pre: Vehicle	BMS score	6	Mice	Thoracic	Female	C57BL/6J
22445934	*all groups*	BBB score	21	Rat	Lumbar	Female	Sprague–Dawley
23544088	100kydn	Grooming score	34	Rat	Cervical	Female	Long–Evans
	12.5 mm	Grooming score	32	Rat	Cervical	Female	Long–Evans
	6.25 mm	Grooming score	10	Rat	Cervical	Female	Long–Evans
	75kydn	Grooming score	58	Rat	Cervical	Female	Long–Evans
32735618	Drug: Female	BMS score	10	Mice	Thoracic	Both	C57BL/6J
	Drug: Male	BMS score	9	Mice	Thoracic	Both	C57BL/6J
	Vehicle: Female	BMS score	10	Mice	Thoracic	Both	C57BL/6J
	Vehicle: Male	BMS score	9	Mice	Thoracic	Both	C57BL/6J

BBB, Basso, Beattie, and Bresnahan Locomotor Scale; BMS, Basso mouse scale open-field score.

### Comparison of published and IAD experimental groups

**FIG. 3.**
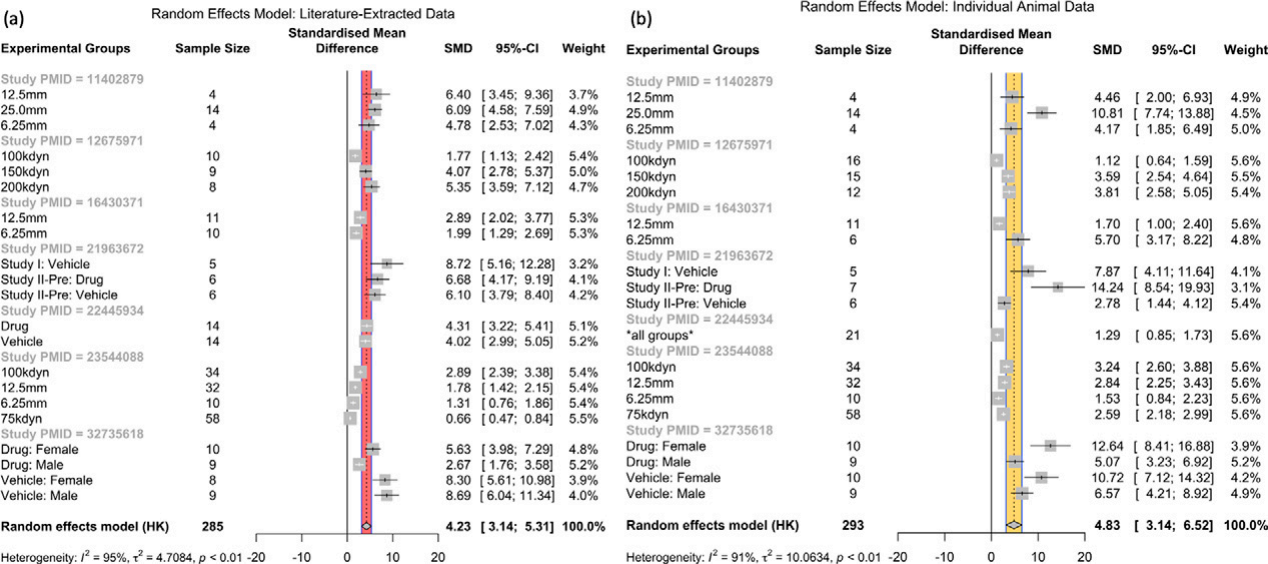
Identical random-effects models (HK) run on both the literature-extracted and individual animal data. **(a)** Random-effects meta-analysis of 7 publications with 21 experimental groups from the literature-extracted data indicating beneficial effect sizes (SMD: 4.23; 95 % CI: [3.14; 5.31]) with significant heterogeneity (*I*^2^ = 95%, *τ*^2^ = 4.7084, *p* < 0.01). **(b)** Random-effects meta-analysis of corresponding individual animal data indicated similar results with beneficial effect sizes (SMD: 4.83; 95% CI: [3.14; 6.52]) and significant heterogeneity (*I*^2^ = 91%, *τ*^2^ = 10.0634, *p* < 0.01). CI, confidence interval; HK, Hartung–Knapp; SMD, standardized mean difference.

Random-effects meta-analysis on 21 groups from LED and the 20 groups from the IAD are depicted in Figure 3a. Random-effects model (HK) yielded an SMD of 4.23 (95% CI: [3.14; 5.31]) showing beneficial effect sizes across the groups in the LED. Expected between-study heterogeneity was confirmed by heterogeneity analysis (*I*^2^ = 95%, *τ*2 = 4.7084, *p* < 0.01). An identical meta-analysis model on the corresponding IAD is shown in Figure 3b. The random-effects model on IAD revealed a larger SMD of 4.83 (95% CI: [3.14; 6.52]) but a similar pattern to LED. Significant heterogeneity was detected in IAD (*I*^2^ = 91%, *τ*^2^ = 10.0634, *p* < 0.01).

### Subgroup analysis

**FIG. 4.**
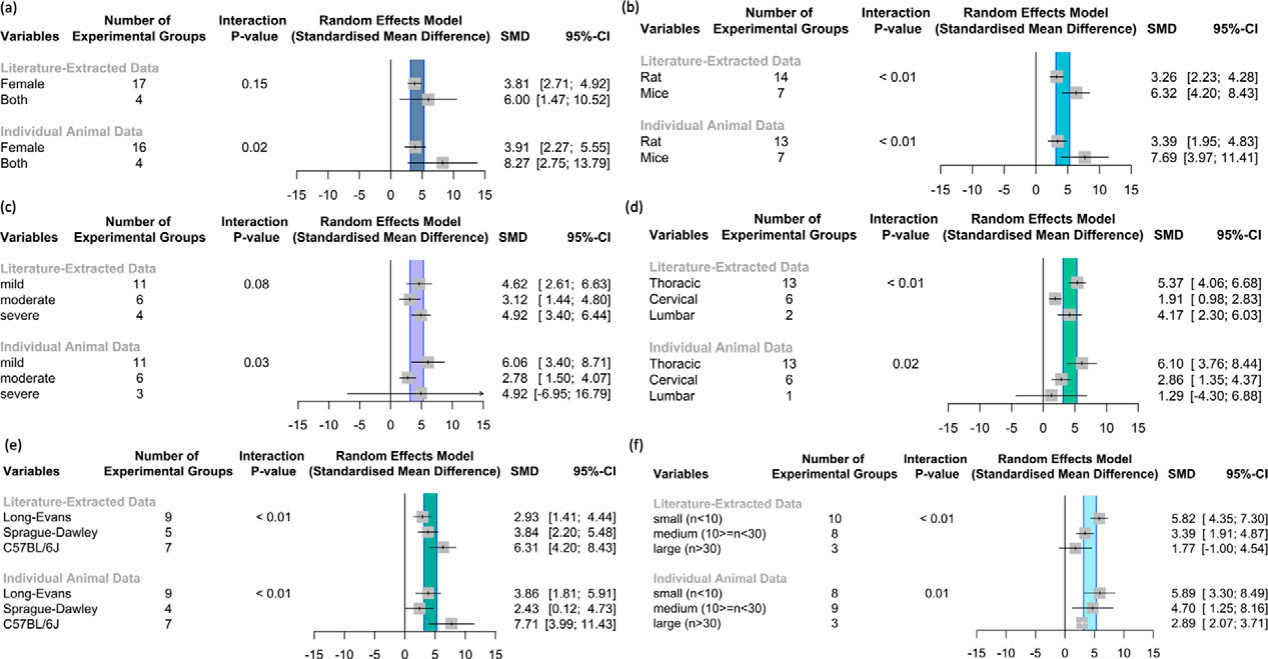
A stratified analysis on subgroups was performed to assess impact of various factors. **(a)** Subgroup analysis on animal sex revealed a statistically significant impact of animal sex on the IAD but not literature-extracted data. **(b)** Stratification by animal type showed significance in both literature-extracted and IAD meta-analyses. **(c)** Injury severity showed different trends between published and IAD. Severe contusion injuries demonstrated the highest effect size in the literature-extracted data, whereas for the IAD, mild contusion injuries were associated with the highest effect size. **(d)** In the literature-extracted data, cervical injuries had the lowest effect size, whereas in the IAD, lumbar injuries showed the lowest effect size. **(e)** Stratification by animal strain significantly influenced both meta-analyses. In the literature-extracted data, Long–Evans rats exhibited the lowest effect size; however, in the IAD, Sprague–Dawley performed worst. **(f)** Groups with a smaller sample size were shown to have a larger effect size, and groups with a larger sample size had smaller effect sizes in both the literature-extracted and individual animal data. IAD, individual animal data.

To assess the impact of various factors within both meta-analyses, we performed a stratified analysis on the subgroups as demonstrated in Figure 4. When assessing the LED, stratification for the animal type (*p* < 0.01, Fig. 4b), level of injury (*p* < 0.01, Fig. 4d), animal strain (*p* < 0.01, Fig. 4e), and sample size (*p* < 0.01, Fig. 4f) accounted for a significant proportion of between-study heterogeneity. For the IAD, all stratifications accounted for statistically significant proportions of heterogeneity; however, the LED did not confirm this for animal sex (Fig. 4a) and injury severity (Fig. 4c).

Although we observed comparable SMD values for some stratifications, absolute numbers of effect sizes did not match across the majority of the stratifications. For injury severity (Fig. 4c), level of injury (Fig. 4d), and animal strain (Fig. 4e), we did not observe comparable trends in the reported effect sizes. Within LED, severe contusion injuries showed the highest effect size (Fig. 4c, SMD 4.92 [95% CI: 3.40; 6.44]), whereas for the IAD, mild contusion injuries had the highest effect size (Fig. 4c, SMD: 6.06 [95% CI: 3.40; 8.71]). Cervical injuries revealed the lowest effect size within the LED (Fig. 4d, SMD: 1.91, 95% CI: [0.98; 2.83]). In IAD, the lowest effect size was detected for lumbar injuries (SMD: 1.29 [95% CI: −4.30; 6.88]). Within the stratification for animal strain, Long–Evans rats showed the lowest effect size in the LED (Fig. 4e; (SMD: 2.93 [95% CI: 1.41; 4.44]); however, Sprague–Dawley rats performed worst in the IAD (SMD: 2.43 [95% CI: 0.12; 4.73]). Figure 4 shows a side-by-side comparison of the subgroup analyses for the literature-extracted and individual animal data. The seperated analyses can be viewed in the supplementary material: see Supplementary Figure S1 for LED and Supplementary Figure S2 for IAD.

## Discussion

We performed a matched meta-analysis to directly compare IAD with their literature-reported summaries. We observed notable mismatches between the LED and the IAD in the number of experimental groups, reported number of animals per group, and reported recovery effect sizes. Notably, IAD analysis had smaller error bars, reflecting the higher power than LED. This echoes work in the clinical literature reporting that individual participant data meta-analysis improves sensitivity for detecting true effects over LED.^6,17,18,37^

Starting with publicly available IAD in the ODC-SCI, we identified 25 matching SCI articles reporting results derived from the same cohort. We then applied the CAMARADES meta-analysis data extraction protocol to the full text (Fig. 2). We observed large differences in the number of experimental groups, number of animals per group, and group labels. Focused analysis on the most commonly used injury models and behavioral outcome measurement reduced the pool from 25 to 7 publications and reduced the total number of animals for the LED by 83% (from *n* = 1841 to *n* = 285), yielding a more homogenous dataset for analysis. Subsequent comparisons of LED with IAD revealed the impact of reporting bias. Our analysis revealed notable effect size differences between LED and IAD analyses (Fig. 4). For example, IAD analysis uncovered significant sex differences that did not reach significance in the LED meta-analysis. In addition, LED and IAD nominated discrepant effects of strain on outcome.

Stratification by injury severity revealed largest effect sizes for mild contusion injuries in IAD, confirming a common biological assumption of SCI researchers that greater sparing provides a better substrate for recovery. However, the LED nominated severe contusions as having the largest effect size, a counterintuitive finding from a clinicopathological standpoint. We also observed distinct trends for neurobehavioral recovery depending on the segmental level of injury, with LED analysis nominating cervical SCI has having smallest effect sizes, whereas IAD nominated lumbar SCI as having the smallest effect sizes. Results also suggested that groups with smaller sample sizes had larger effect sizes, which has been previously interpreted to reflect that researchers terminate studies early upon seeing large effects, even though these effects likely reflect statistical noise rather than true effect sizes.^18,38^ This analysis highlights potential inflation of effect sizes in inadequately powered small studies. Prior studies have shown that smaller sample sizes may lead to overoptimistic conclusions, with effect sizes appearing higher than they would be with adequate sample size.^39^ It is noteworthy that LED analyses often produced unrealistically small error bars, when compared with the IAD from the same subjects (Fig. 4f).

The results provide opportunities for translational dialog with clinical meta-analysis, the major tool for ranking evidence in clinical decision support.^7^ The clinical classes of evidence (CoE) grading system places meta-analysis of randomized controlled clinical trials as the highest grade of clinical evidence (Class I).^40^ Pre-clinical and mechanistic studies are ranked as the weakest evidence, meaning that pre-clinical research has little impact on clinical decision-making. The clinical CoE system is so different from the pre-clinical literature definition of “robust effects” that it is difficult for clinicians to gauge pre-clinical research and make objective, informed decisions about which therapies should advance into clinical studies. As a result, translation may occur in a haphazard manner based on journal impact and citations rather than rigor and reproducibility. Prior reports suggest that most pre-clinical therapies reported in high-impact journals cannot be independently replicated,^41^ leading to the perception that pre-clinical literature lacks standardization and relies on underpowered studies with questionable evidentiary basis.^42^ Yet, pre-clinical research remains the majority of biomedical scientific literature and represents the majority of the global research investment in biomedicine. For example, a recent report from the U.S. NIH suggests that it spends over 50% of its budget on basic science and continues to view pre-clinical science as the bedrock of biomedical discovery.^43^ Yet, considerably less resources are dedicated to assessing the reproducibility of these discoveries, resulting in what some have called “canonization of false facts.”^44^

Specifically for the field of pre-clinical SCI research, the current study confirms that publications as a final “scientific product” contain highly selected datasets. Reporting selective subsets of data may unintentionally sway results to more desirable outcomes, potentially obscuring true effect sizes. These findings place an emphasis on the need for better transparency in reporting, including sharing individual subject data, instead of letting it become dark data, data inaccessible to the research community.^12,45^ Without access to IAD, it is difficult to evaluate which experimental therapeutics are best candidates for clinical translation.

This work has some limitations. Because of the 37 subjects with missing experimental grouping information, we could only partially register individual subjects to their corresponding representation in published articles. This may have affected the reported neurobehavioral recovery, as attrition bias (e.g., the removal of outliers) has been reported to significantly modify results even if only very few animals had been removed from a particular experimental group.^46^ In addition, our analysis cannot discriminate whether the chosen outcome parameters were truly justified to detect the best possible effect in all the published experiments. This may influence the results since outcome testing modalities need to be chosen carefully with respect to the specific injury model, severity, and location.^47^

**FIG. 5.**
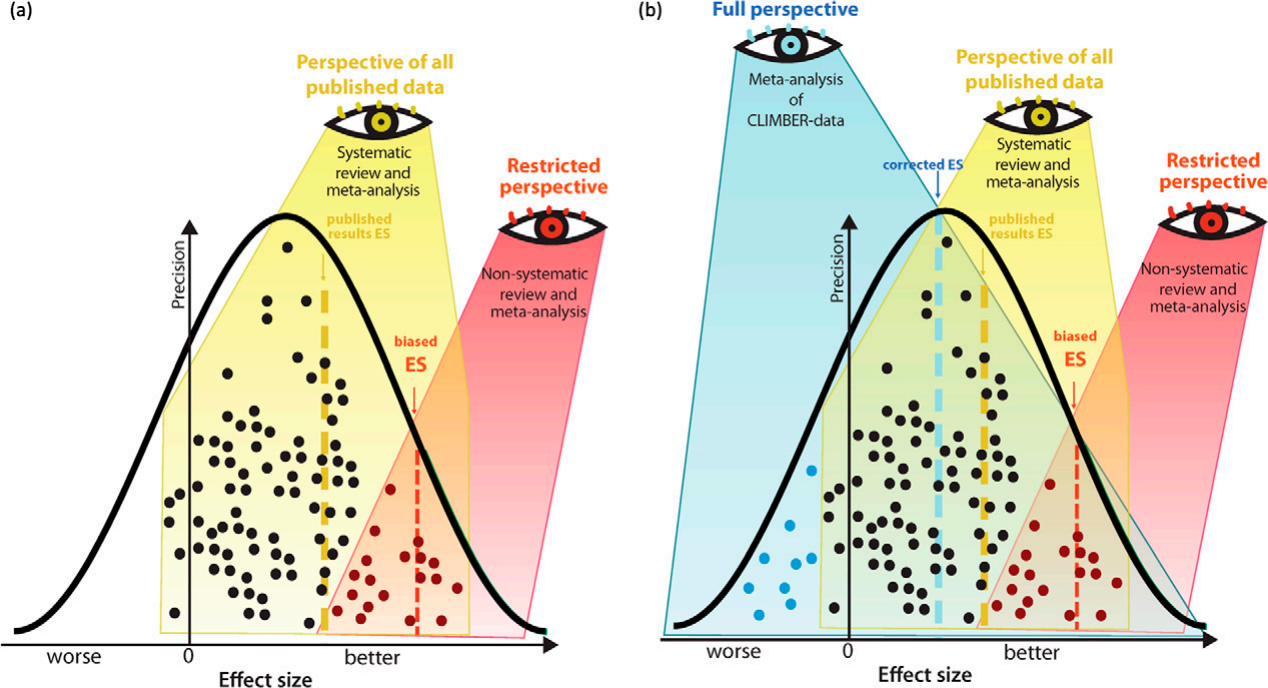
Traditional meta-analysis versus meta-analysis of CLIMBER data. **(a)** Illustration of the traditional approach to meta-analysis. **(b)** Illustration of the CLIMBER meta-analysis with enhanced precision and corrected effect sizes for more accurate results. Each point represents an individual animal.

Together, the results demonstrate the utility of combining IAD analysis and traditional literature-sourced meta-analysis to explore effect size reproducibility in SCI. By combining IAD with LED, future work will be able to gain additional insights on findings across diverse studies, helping to improve the predictive value for clinical translation (Fig. 5). Augmented, combinatorial analysis strategies may allow for higher evidence levels of pre-clinical findings. The results underline the added value of the FAIR data sharing (ODC-SCI) to better understand missing data to improve robustness of translation.

## Abbreviations Used

ARRIVE: Animal Research: Reporting of In Vivo Experiments
BMS: Basso Mouse Scale
BBB: Basso, Beattie, and Bresnahan Locomotor Scale
CAMARADES: Collaborative Approach to Meta-Analysis and Review of Animal Data in Experimental Studies
HK: Hartung—Knapp
IAD: individual animal data
LED: literatureextracted data
ODC-SCI: Open Data Commons for Spinal Cord Injury
ODC-TBI: Open Data Commons for Traumatic Brain Injury
SCI: spinal cord injury
SMD: Standardized mean differenc
