# Supplementary material for: Effect-Size Discrepancies in Literature Versus Raw Datasets from Experimental Spinal Cord Injury Studies: A CLIMBER Meta-Analysis
Source: Neurotrauma Rep. 2024 Jul 16;5(1):686–98. doi: 10.1089/neur.2024.0038 (PMC11271150; doi:10.1089/neur.2024.0038)
Supplement: Supplementary Table S1 [file neur.2024.0038_ioriosupplementary_table1.pdf]

Supp. Table 1. Literature-Extracted Groups Prior to Final Exclusion

PMID	Cohort Label	Neurobehavioral Score	Sample Size
11402879	12.5mm	BBB Score	4
	25.0mm	BBB Score	14
	6.25mm	BBB Score	4
12675971	100kdyn	BBB Score	10
	150kdyn	BBB Score	9
	200kdyn	BBB Score	8
16430371	12.5mm	Grooming Score	11
	6.25mm	Grooming Score	10
21963672	Study I: Vehicle	BMS Score	5
	Study I: Drug	BMS Score	5
	Study II-Post: Drug	BMS Score	6
	Study II-Post: Vehicle	BMS Score	6
	Study II-Pre: Drug	BMS Score	6
	Study II-Pre: Vehicle	BMS Score	6
22445934	Drug	BBB Score	14
	Vehicle	BBB Score	14
23544088	100kdyn	Grooming Score	34
	12.5mm	Grooming Score	32
	6.25mm	Grooming Score	10
	75kdyn	Grooming Score	58
32735618	Drug: Female	BMS Score	10
	Drug: Male	BMS Score	9
	Drug: Male	BMS Subscore	9
	Vehicle: Female	BMS Score	8
	Vehicle: Male	BMS Score	9
		**Total N**	**311**
