# Supplementary material for: Effect-Size Discrepancies in Literature Versus Raw Datasets from Experimental Spinal Cord Injury Studies: A CLIMBER Meta-Analysis
Source: Neurotrauma Rep. 2024 Jul 16;5(1):686–98. doi: 10.1089/neur.2024.0038 (PMC11271150; doi:10.1089/neur.2024.0038)
Supplement: Supplementary Table S2 [file neur.2024.0038_ioriosupplementary_table2.pdf]

Supp. Table 2. Individual Animal Groups Prior to Final Exclusion

PMID	Cohort Label	Neurobehavioral Score	Sample Size
11402879	12.5mm	BBB Score	4
	25.0mm	BBB Score	14
	6.25mm	BBB Score	4
12675971	100kydn	BBB Score	16
	150kydn	BBB Score	15
	200kydn	BBB Score	12
16430371	12.5mm	Forelimb OpenField Score	11
	6.25mm	Forelimb OpenField Score	6
21963672	*unknown*	BMS Score	11
	Study I: Vehicle	BMS Score	5
	Study II-Pre: Drug	BMS Score	7
	Study II-Pre: Vehicle	BMS Score	6
22445934	*all groups*	BBB Score	21
23544088	100kydn	Grooming Score	34
	12.5mm	Grooming Score	32
	6.25mm	Grooming Score	10
	75kydn	Grooming Score	58
32735618	Drug: Female	BMS Score	10
	Drug: Male	BMS Score	9
	Vehicle: Female	BMS Score	10
	Vehicle: Male	BMS Score	9
		**Total N**	304
